# Limited Cross-Complementation Between *Haloferax volcanii* PilB1-C1 and PilB3-C3 Paralogs

**DOI:** 10.3389/fmicb.2019.00700

**Published:** 2019-04-24

**Authors:** Georgio Legerme, Mechthild Pohlschroder

**Affiliations:** Department of Biology, University of Pennsylvania, Philadelphia, PA, United States

**Keywords:** *Haloferax volcanii*, pilus biosynthesis, pilin, archaea, type IV pili, adhesion, biofilm formation

## Abstract

Type IV pili are evolutionarily conserved cell surface filaments that promote surface adhesion and cell aggregation providing bacteria and archaea protection from a variety of stress conditions. In fact, prokaryotic genomes frequently contain several copies of the core biosynthesis genes, *pilB* and *pilC*, encoding an ATPase and membrane anchor, respectively. Recent phylogenetic analyses suggest that in haloarchaea, a subset of *pilB-C* paralogs, such as the *Haloferax volcanii pilB1*-*C1*, were gained *via* horizontal transfer from the crenarchaea, while the co-regulated type IV pilus subunits, the pilins, evolved by duplication, followed by diversification of the ancestral euryarchaeal pilins. Here, we report the identification of an *H. volcanii pilB1* transposon mutant that exhibits an adhesion defect in defined media. A similar defect observed in an *H. volcanii* ∆*pilB1-C1* strain can be rescued by expressing *pilB1-C1 in trans*. However, these proteins cannot rescue the severe adhesion defect of a previously reported ∆*pilB3-C3* strain. Conversely, *pilB3-C3,* which are not predicted to have been laterally transferred, expressed *in trans* can rescue the adhesion defect of a ∆*pilB1-C1* strain. This cross-complementation supports the proposed hybrid origin of the operon containing *pilB1-C1* and shows that at least certain euryarchaeal PilB paralogs can work with different pilin sets. Efficient recognition of the euryarchaeal pilins by the crenarchaeal PilB1-C1 may have required some degree of pilin modification, but perhaps the modifications were minor enough that PilB3 recognition of these pilins was not precluded, resulting in modular evolution and an extensive combinatorial diversity that allows for adaptation to a variety of stress conditions and attachment to varied surfaces.

## Introduction

Type IV pili are thin filament-like protein complexes that extend from the cell surface and display a great deal of versatility in the roles they play in various cellular processes. As they are found in species that are representative of most bacterial and archaeal phyla, these structures are considered to be evolutionarily ancient (Pohlschroder et al., 2011; Hospenthal et al., 2017; Chaudhury et al., 2018). Among the functional roles that these ancient structures commonly play in species from both prokaryotic domains are promoting adherence to biotic and abiotic surfaces, facilitating close intracellular associations, and mediating the formation and maturation of biofilms; moreover, a type IV pilus-related cell surface structure known as the archaealla drives swimming motility in archaea (Albers and Jarrell, 2018).

Type IV pili are composed of major and minor pilins, with the major pilin comprising the bulk of the filamentous structure. Before these pilins can be assembled into a type IV pilus, a prepilin peptidase, PibD, in archaea, must cleave off their signal peptides, exposing N-terminal hydrophobic domains in the mature pilins. Interactions between these hydrophobic domains then drive the formation of a hydrophobic central core that serves as a scaffold for assembly of the pilus. In addition to the prepilin peptidase, completing the assembly of a functional pilus requires core components that include PilB, an ATPase that provides the energy required for assembly, and PilC, a multispanning transmembrane protein required for anchoring of the surface filament to the cell membrane (Pohlschroder and Esquivel, 2015; Chaudhury et al., 2018; Pohlschroder et al., 2018). Most prokaryotic genomes encode several *pilB-C* paralogs, many of which are contained in operons that also include pilin genes, indicating that the transcription of paralogs is often co-regulated along with a specific set of pilins (Szabo et al., 2007; Imam et al., 2011; Pohlschroder et al., 2011). Furthermore, many prokaryotic genomes are predicted to encode more than 40 pilins, suggesting that these pilins might be assembled into a variety of type IV pili having a broad range of diverse functional roles to play, likely depending on local environmental conditions. Unfortunately, the specific functional roles played by most of these pilins are not yet known (Szabo et al., 2007; Imam et al., 2011; Losensky et al., 2014; Pohlschroder et al., 2018).

Studies performed in the model euryarchaeon, *Haloferax volcanii*, which encodes five *pil* operons (*pilB1-C1*-*pilB5-C5*) as well as the archaealla (*arl*) operon, have revealed still greater complexity in the diversity of type IV pili in archaea (Szabo et al., 2007). In addition to the *arl* operon, the *pilB3-C3* genes, which are in an operon that does not include pilin-encoding genes, have been studied *in vivo* in some detail (Esquivel et al., 2013, 2016; Esquivel and Pohlschroder, 2014). PilB3-C3 are required for the biosynthesis of pili containing any of the adhesion pilins, PilA[1–6], which are a subset of the 42 pilins encoded by the *H. volcanii* genome, as predicted by *in silico* analysis (Esquivel et al., 2013; Tripepi et al., 2013; Esquivel and Pohlschroder, 2014). Each of these predicted pilins contains a completely conserved pilin H-domain. In fact, we have determined that each of the six adhesion pilins can be assembled into a type IV pilus, and that the pili assembled from each of these adhesion pilins appear to play a distinct role during the early stages of biofilm formation (Esquivel and Pohlschroder, 2014). While no significant adhesion can be observed in an *H. volcanii* ∆*pilA[1-6]* strain during the first 24 hours of incubation, residual adhesion is present when a ∆*pilB3-C3* strain is incubated for that period of time, suggesting that *PilB-C* paralogs can complement the ∆*pilB3-C3* deletion (Esquivel and Pohlschroder, 2014).

To help understand functional and evolutionary relationships between these somewhat disparate biosynthesis systems, Makarova et al. used *in silico* analyses to phylogenetically group the archaeal PilB ATPases into four clades comprised of clade 1 methanogens, clade 2 euryarchaea, including *H. volcanii* PilB3, PilB4 and PilB5, clade 3 archaellum, and clade 4 TACK superphylum (Makarova et al., 2016). In these analyses, predicted pilin genes that are associated with *pilB-C* were also examined. A few haloarchaeal PilB homologs including *H. volcanii* PilB1 and PilB2 were identified to belong to a subclade of clade 4 (clade 4C). However, it was determined that the genes encoding these PilB homologs are associated with genes encoding pilins belonging to the family of pilins generally associated with clade 2 PilB ATPases, leading to the hypothesis that the PilB and PilC components encoded by some euryarchaeal pilus operons were acquired *via* horizontal transfer from the crenarchaea, while the pilin genes associated with these *pilBC* genes are the result of duplication and diversification of ancestral euryarchaeal pilin genes. This proposed hybrid origin led the authors to further propose that the archaeal PilB “can work with different pilin sets resulting in modular evolution and extensive combinatorial diversity” (Makarova et al., 2016).

Here, we report that a screen of a transposon insertion library has led to the identification of an adhesion mutant that has an insertion in *H. volcanii pilB1*. We also determined that a ∆*pilB1-C1* strain has a similar adhesion defect that can be rescued by the expression of *pilB1-C1 in trans*. However, we have also shown that a ∆*pilB1-C1* ∆*pilB3-C3* double mutant does not abolish the residual adhesion observed in the ∆*pilB3-C3* strain, nor can the ∆*pilB3-C3* adhesion defect be rescued by the expression of *pilB1-C1 in trans*. Conversely, *pilB3-C3* expressed *in trans* does rescue the adhesion defect of a ∆*pilB1-C1* strain, suggesting that the major pilin may have undergone some alterations prior to being efficiently recognized by PilB1-C1 after it was acquired through horizontal transfer while the modification of this pilin was not to such a degree that it could not be recognized by PilB3 anymore.

## Materials and Methods

### Reagents

For basic molecular biology procedures, reagents were purchased from New England BioLabs, except for the iProof High-Fidelity DNA polymerase, which was purchased from Bio-Rad. For genomic DNA extraction, the Thermo scientific GeneJET Genomic DNA Purification kit was used while for plasmid purification, kits were purchased from Qiagen. Difco agar and Bacto yeast extract were purchased from Becton, Dickinson. Peptone was purchased from Oxoid. 5-fluoroorotic acid (5-FOA) was purchased from Toronto Research Biochemicals. All other chemicals and reagents were purchased from either Thermo Fisher Scientific or Sigma-Aldrich.

### Strains and Growth Conditions

The plasmids and strains used in this study are listed in Table 1. *H. volcanii* strain H53 and its derivatives were grown at 45°C in liquid (orbital shaker at 250 rpm) or on solid casamino acid (CA) medium supplemented with tryptophan and uracil (both at 50 μg ml^−1^ final concentration). 1.5% agar was used to create solid medium in petri dishes. Strains transformed with pTA963, pGL3, or pRE62 were grown on CA medium supplemented with tryptophan (50 μg ml^−1^ final concentration). FH37, a variant of the H53 strain expressing a chromosomally encoded tryptophan gene, and GL1 were grown on CA medium supplemented with uracil (50 μg ml^−1^ final concentration). For selection of the deletion mutant, 5-FOA was added at a final concentration of 150 μg ml^−1^ in CA medium and uracil was added to a final concentration of 10μgml^−1^. *Escherichia coli* strains were grown at 37°C in NZCYM medium supplemented with ampicillin (200 μg ml^−1^).

**Table 1:** Plasmids and strains.

Plasmid or strain	Characteristic(s)	Reference or source
Plasmids		
pTA131	Amp^r^; *pyrE2* under a ferredoxin promoter	(Allers et al., 2004)
pTA963	Amp^r^; *pyrE2* and *hdrB* markers, inducible	(Allers et al., 2010)
	*ptna* promoter	
pGL2	pTA131 carrying chromosomal *pilB1-C1*	This study
	flanking regions	
pGL3	pTA963 containing *pilB1-C1His*	This study
pRE62	pTA963 containing *pilB3-C3His*	(Esquivel and Pohlschroder, 2014)
*E. coli* strains		
DH5alpha	F-80d*lacZ*ΔM15 Δ(*lacZYA-argF*)*U169 recA1*	Invitrogen
	*endA hsdR17*(rK- mK-) *supE44 thi-1 gyrA relA1*	
DL739	MC4100 *recA dam-13::*Tn9	(Blyn et al., 1990)
*H. volcanii* strains		
H53	Δ*pyrE2* Δ*trpA*	(Allers et al., 2004)
FH37	H53 Δ*pyrE2*	(Abdul Halim et al., 2015)
RE43	H53 Δ*pilA1* Δ*pilA2* Δ*pilA3* Δ*pilA4* Δ*pilA5* Δ*pilA6*	(Esquivel et al., 2013)
GL1	H295 *pilB1*::*tn*	This study
GL 20	H53 Δ*pilB1-C1*	This study
GL 21	H53 Δ*pilB1*-*C1*Δ*pilB3-C3*	This study
GL 22	H53 Δ*pilB1-C1* containing pTA963	This study
GL 23	H53 Δ*pilB1*-*C1*Δ*pilB3*-*C3* containing pTA963	This study
GL 24	H53 containing pGL3	This study
GL 25	H53 Δ*pilB1-C1* containing pGL3	This study
GL 26	H53 Δ*pilB1*-*C1*Δ*pilB3*-*C3* containing pGL3	This study
GL 27	H53 Δ*pilB3*-*C3* containing pGL3	This study
GL 28	H53 containing pRE62	This study
GL 29	H53 Δ*pilB1-C1* containing pRE62	This study
GL 30	H53 Δ*pilB1*-*C1*Δ*pilB3*-*C3* containing pRE62	This study

### Growth Curves

Growth curves were generated using a Biotek PowerWaveX2 microplate spectrophotometer. *H. volcanii* strains were first incubated in 5-ml liquid cultures in CA medium supplemented with tryptophan and uracil with continuous shaking at 45°C, until suitable OD_600_ values (0.2–0.5) were reached. Approximately 2 μl of each culture (adjusted for OD_600_ differences) was then transferred into 198 μl of fresh CA medium supplemented with tryptophan and grown to stationary phase, with OD_600_ recordings taken every 30 min.

### Surface Adhesion Transposon Mutant Screening Assay

Surface adhesion to 96-well plate wells was used to screen transposon mutants and was previously developed, modifying the air-liquid interface (ALI) assay described by O’Toole (O’Toole et al., 1999; Legerme et al., 2016).

To improve consistency, subsequent adhesion assays employing 96-well plates for individual adhesion mutants were modified. Liquid cultures of volume 5 ml were grown to exponential phase in either CA medium supplemented with tryptophan or both tryptophan and uracil. After a second transfer into 5-ml liquid media, 200 μl of this liquid culture was then placed in wells at 45°C with no agitation for 30 h. After the incubation period, the cell adhesion was determined as described above using the Biotek reader.

### Cover Slip Adhesion Assay

*H. volcanii* surface adhesion to a plastic cover slip was assayed using a modified ALI assay as described by Esquivel et al. (2016). Briefly, 3 ml of culture in CA medium supplemented with uracil was grown to an optical density at 600 nm (OD_600_) of ~0.3 and incubated in each well of a covered 12-well plate. Plastic cover slips (22 mm× 22 mm; 0.19–0.25 mm thick) from Fisher Scientific (Hampton, NH, USA) were inserted into each well at a 90° angle and incubated at 45°C without shaking. To visualize adhering cells, cover slips were removed and fixed with 2% (*v/v*) acetic acid for 3 min; coverslips were air-dried and then stained in 0.1% (*w/v*) crystal violet (CV) solution for 10 min. The coverslips were then washed with distilled water, air-dried, and examined using light microscopy.

### Identification of Transposon Integration Sites by Next-Generation Sequencing

A Nextera Kit (Illumina; San Diego, CA, USA) was used to prepare the motility and/or adhesion mutant DNA for genome sequencing using a 300-cycle micro v2 MiSeq (Illumina). DNA was purified with the Zymo DNA clean and concentrator Kit (Irvine, CA, USA) and diluted in sterile water to a concentration of 10 ng/μl. Mutant genomic reads were assembled and mapped to the reference transposon sequence using the program, Geneious (Biomatters Ltd; Auckland, New Zealand), and the genomic sequence flanking the transposon was used to identify the transposon-disrupted gene through the National Center for Biotechnology Information’s (NCBI) Basic Local Alignment Search Tool (BLAST) (Altschul et al., 1997). Gene annotations were obtained from NCBI unless otherwise noted.

### Generation of Chromosomal Deletions

Chromosomal deletions of *hvo_0619* and *hvo_0620* were generated in the H53 wild-type strain and RE43 deletion strains using a homologous-recombination-based (pop-in pop-out) method previously described (Allers and Ngo, 2003). Plasmid constructs were generated using overlap PCR with modifications (Tripepi et al., 2010) using primers listed in Table 2. To confirm that the chromosomal replacement event occurred at the proper location on the chromosome, genomic DNA isolated from colonies derived using these techniques was screened by PCR using two primer pairs, one hybridizing at the 5′ end of *hvo_0619* and 3′ end of *hvo_0620* and the other hybridizing to genomic sequences flanking the deletion (Table 2). The identities of the PCR products were verified by DNA sequencing.

**Table 2:** Primers used for PCR amplification.

Primer name/Sequence (5′-3′)	Target sequence
FWPILB1KO	
AAGCTAGGATCCTCCAGATACGCGCCG	700 bp upstream of *hvo_0620* start codon, extension toward gene
PilB1C1KO-R2	
GATATCTCTAGACGGTTCGAGTCGTGTTG	700 bp downstream of *hvo_0619* stop codon, extension toward gene
PilB1C1KO-OV-F	
CTCAGCAAAGCACGGTATATGAGCGCCACCCG	15 bp upstream of stop codon, extension away from gene
PilB1C1KO-OV-R	
CGGGTGGCGCTCATATACCGTGCTTTGCTGAG	15 bp downstream of start codon, extension away from gene
ExpVec-PilB1C1-F	
GATATCCATATGCCTACCCAAGGC	At the start codon of *hvo_0620*
ExpVec-PilB1C1-R	
GATATCGGATCCTCAGTGGTGGTGGTGGTGGTGGGCGCTAGCTATCAGGCCCACCCC	At the end of *hvo_0619* before overlap with *hvo_0618*

## Results and Discussion

### Identification of *pilB1::tn* Adhesion Mutant Through Screen of Transposon Insertion Library

The establishment of an *H. volcanii* transposon insertion library and the modification of the 96-well air-liquid interface (ALI) assay for use with high salt conditions resembling those that haloarchaea inhabit have recently allowed for efficient screening for adhesion mutants in this model haloarchaeon (Kiljunen et al., 2014; Legerme et al., 2016). The fixing and staining of cells in 96-well plates results in observable rings on the well surface at the air-liquid interface, which allows a determination of the relative efficiency with which cells adhere. Cells harboring mutations that result in a significant decrease in the strength of surface adhesion, but do not affect growth, can be readily identified since the rings they form stain less intensely. To help differentiate between normal and deficient adhesion, a wild-type strain and Δ*pilA*[*1-6*], an adhesion-deficient mutant that lacks major pilins (Esquivel et al., 2013), were used as standards in these assays. Genome sequencing was then used to identify the transposon insertion location.

**Figure 1:**
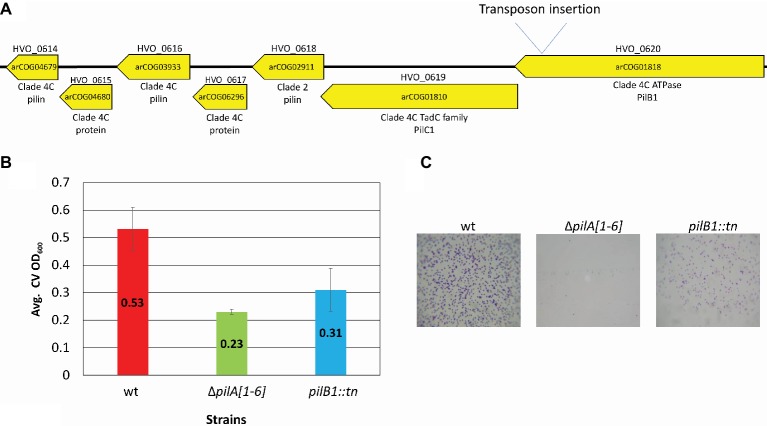
*pilB1::tn* exhibits a severe adhesion defect. **(A)** The location of the *pilB1*::*tn* insertion between base pair 550,339 and 550,340 was determined by whole genome sequencing. Genes of the *pilB1* operon labeled using the *H. volcanii* locus tag (listed on top of gene), archaeal clusters of orthologous genes (arCOG) (listed within gene), and the clade that they are classified under as determined by Makarova et al. (2016) are listed below the gene. Genes in this operon are either part of clade 4C or clade 2. **(B)** Using the 96-well surface adhesion assay described in Legerme et al. (2016), the averages ± standard deviations were determined to compare adhesion of three biological replicates of wt (FH37), Δ*pilA*[*1-6*], and *pilB1::tn* strains grown in casamino acid (CA) media. This showed reduced relative adhesion of *pilB1::tn*. **(C)** Representative adhesion to plastic coverslips incubated for 12 h in exponential phase culture in wells of 12-well plates and subsequent staining of attached cells as described previously (Esquivel et al., 2013) confirmed the adhesion defect phenotype observed in **(B)**. (×500 magnification) (*n* = 3).

Employing these methods, we were able to identify an adhesion mutant that has a transposon insertion in *pilB1*, a gene that encodes a paralog of PilB, an ATPase required for pilus assembly (Figure 1A). While the adhesion defect of this transposon mutant is not as severe as that of Δ*pilA*[*1-6*], the 96-well plate ALI assay indicates that the strength of adhesion of *pilB1::tn* cells is less than that of the wild-type (Figure 1B).

Additionally, a surface adhesion assay using immersed coverslips confirmed this phenotype and its strength relative to wild-type and the Δ*pilA*[*1-6*] cells (Figure 1C). This result is the first indication of an *H. volcanii* PilB paralog other than PilB3 being involved in surface adhesion.

However, due to the ambiguities that can result from a transposon insertion, which include the possibility that the insertion disrupts the transcription of the whole operon, a deletion strain to confirm the phenotype observed in the *pilB1*::*tn* mutant was generated.

### Construction and Characterization of *ΔpilB1-C1* Mutant

**Figure 2:**
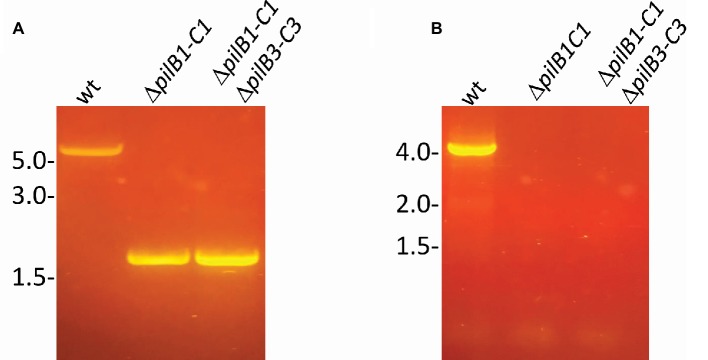
Confirmation of chromosomal *pilB1-C1* deletion. PCR amplification was performed using primers **(A)** against the flanking regions located approximately 700 bp upstream and 700 bp downstream of *pilB1-C1* and **(B)** specific for the *pilB1* and *pilC1* genes. The template DNA used was isolated from the wild-type or Δ*pilB1-C1* strain.

To confirm the importance of PilB1 for adhesion, a deletion mutant was created. Since *pilB1* and *pilC1* overlap by four amino acids and are functionally paired in many species, we used homologous recombination to generate an *H. volcanii* deletion strain lacking both genes (Δ*pilB1-C1*), except for the last four nucleotides of *pilC1*, as these nucleotides also overlap with the adjacent gene, the predicted pilin gene *hvo_0618*, (Allers and Ngo, 2003; Takhar et al., 2013). We confirmed the deletion by PCR using a forward primer homologous to sequences lying just inside *pilB1* and a reverse primer homologous to PilC1 sequences starting four nucleotides into *pilC1* (Figure 2; Table 1) as done previously for all other deletion strains, such as Δ*pilB3-C3* used in this study (Esquivel et al., 2014).

The Δ*pilB1*-*C1* strain was then characterized using a modified version of the 96-well surface adhesion assay that was used to screen the transposon insertion library. Since the number of analyzed strains was strongly decreased, they could be grown in shaking culture tubes to exponential phase (OD_600_ 0.3) and then be incubated in wells for adhesion, eliminating the need for agitation of the 96-well plate. Although agitation increased aeration and fostered nutrient homogeneity within the media, and thus promoted growth (Juergensmeyer et al., 2007), the turbulence caused by agitation increased shear stress in the wells, reducing adhesion efficiency after initial attachment and perhaps even initial attachment (Moreira et al., 2013). Following static incubation for 30 h, adhesion levels, as determined by crystal violet (CV) absorbance, were more precise, and the differences in adhesion between cultures over time could be determined more effectively, especially given that the initial inoculum was less variable.

**Figure 3:**
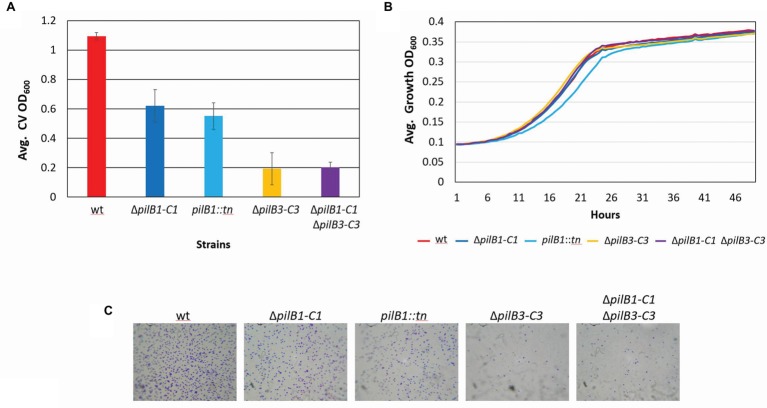
*H. volcanii* Δ*pilB1-C1* has an intermediate adhesion defect similar to that of *pilB1::tn*; however, PilB1-C1 is not responsible for the residual attachment of Δ*pilB3-C3*. **(A)** Using the further modified 96-well surface adhesion assay described in the Materials and Methods with stationary 30-h incubation, *H. volcanii* wild-type (H53), Δ*pilB1-C1*, *pilB1::tn*, Δ*pilB3-C3*, and Δ*pilB1-C1*Δ*pilB3-C3* strain adhesion was quantified by measuring the OD_600_ of the stain released in methanol. Three biological replicates with three technical replicates each were completed, and averages (± s.d.), correlating to adhesion, were determined showing similarly reduced adhesion of Δ*pilB1-C1* and *pilB1::tn.* **(B)** As described in the Materials and Methods, 49-h growth curves were generated for each strain with average growth OD_600_ calculated and graphed for three biological replicates of each strain, each with three technical replicates showing similar growth patterns.**(C)** Adhesion profiles of aforementioned strains were determined by incubation of plastic coverslips in exponential phase culture for 24 h in wells of 12-well plates. The images shown are representative of six replicates and are consistent with the results observed in **(A)**.

**Figure 4:**
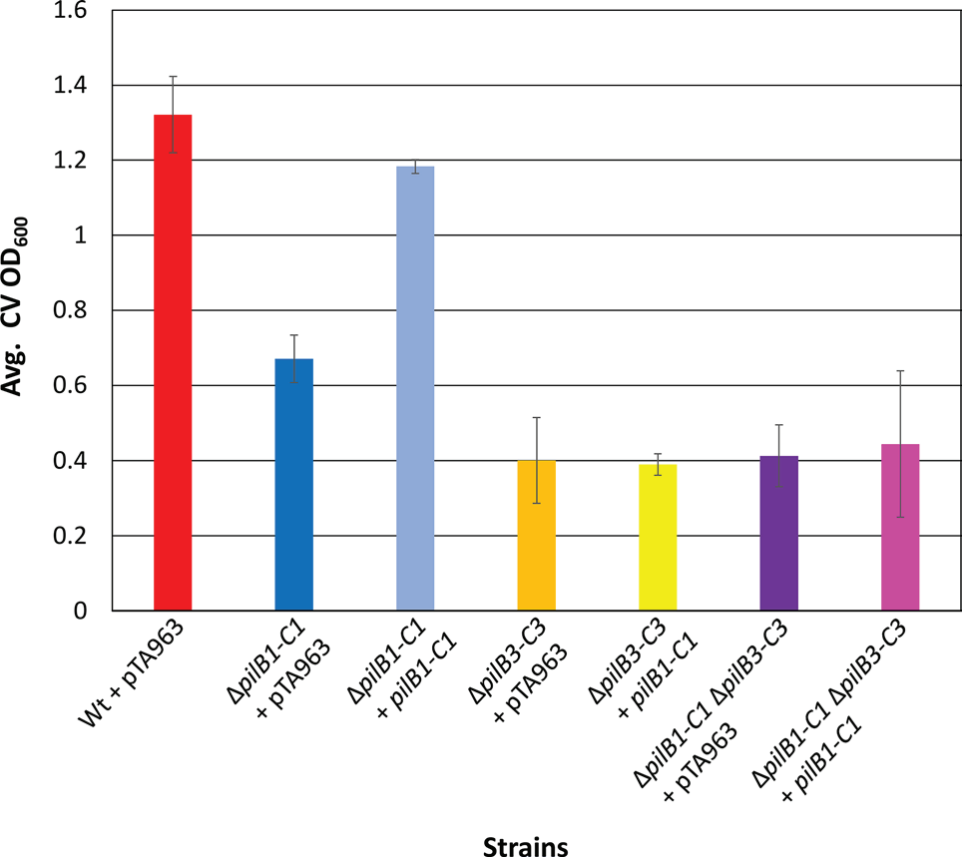
*pilB1-C1* rescues adhesion defect of the Δ*pilB1-C1* strain to near wild-type adhesion but does not complement Δ*pilB3-C3* or Δ*pilB1-C1*Δ*pilB3-C3* adhesion defects. Using the 96-well ALI assay described in Figure 3A, averages (± s.d.) of adhesion quantified by determination of CV OD_600_ for three biological replicates, with three technical replicates each, of wild-type + pTA963 and Δ*pilB1-C1*, Δ*pilB3-C3*, and Δ*pilB1-C1*Δ*pilB3-C3* strains transformed with pTA963 or pTA963 encoding his-tagged *pilB1-C1*; all transformed strains were grown in CA media. Similar adhesion levels between wild-type + pTA963 and Δ*pilB1-C1* + *pilB1-C1* are shown but those for Δ*pilB3-C3* + *pilB1-C1* and Δ*pilB1-C1*Δ*pilB3-C3* + *pilB1-C1* are still reduced.

Using this surface adhesion assay to characterize strains grown in semi-defined media, it was shown that, like the *pilB1::tn* strain, Δ*pilB1-C1* also has an intermediately reduced adhesion phenotype relative to the wild-type strain. Interestingly, under these conditions, the *pilB1::tn* strain consistently had an adhesion phenotype that was slightly more deficient than that of Δ*pilB1-C1* (Figure 3A), perhaps because the transposon insertion in *pilB1* may have affected the transcription of all genes in the operon containing *pilB1* as opposed to the effect of completely deleting two specific genes in that operon. The expression of *pilB1-C1his in trans* under the control of an inducible *trp* promoter rescues the Δ*pilB1-C1* phenotype, underscoring the fact that the adhesion defect of this strain is indeed due to the absence of these pilus biosynthesis genes (Figure 4).

Additional characterization of these mutant strains included the generation of growth curves to determine whether deletion or disruption of *pilB1-C1* had a significant effect on strain growth relative to the wild-type to ensure that reduced adhesion was not a result of diminished growth but rather solely a result of the functional absence of these components of the pilus biosynthesis machinery. Ultimately, we determined that the *pilB1::tn* and Δ*pilB1-C1* strains had growth rates similar to that of the wild-type (Figure 3B). Interestingly, *pilB1::tn* has slightly delayed growth during exponential phase compared to Δ*pilB1-C1* and wild-type, and, although this deficiency is overcome during stationary phase, this could also explain the slight difference in adhesion phenotypes observed in the *pilB1::tn* and Δ*pilB1-C1* strains.

Using the 12-well surface adhesion assay with coverslips, we confirmed the adhesion defects of the mutant strains as the quantity of cells in the adhesion profile of each strain correlated well with the CV quantification results obtained with the 96-well plate assays (Figure 3C). While previous results showed that some pilin mutant strains have phenotypes that include early microcolony formation, it should be noted that neither *pilB1::tn* nor Δ*pilB1-C1* have increased early microcolony formation under the conditions tested (Esquivel et al., 2013).

### Analysis of PilB1-C1 Involvement in PilB3-C3-Dependent Adhesion

It was previously shown that, unlike Δ*pilA*[*1-6*], which lacks residual attachment, cells lacking the *pilB3-C3* biosynthesis genes, which are required for PilA[1*–*6] pili assembly exhibit residual adhesion (Esquivel and Pohlschroder, 2014). It is possible that PilB1-C1 are responsible for the residual adhesion of the Δ*pilB3-C3* strain through the assembly of a subset of PilA[1–6] pili. To test this hypothesis, we deleted *pilB1-C1* in the Δ*pilB3-C3* strain generating the double mutant, Δ*pilB1-C1*Δ*pilB3-C3.* Δ*pilB1-C1-*Δ*pilB3-C3* has the same adhesion phenotype as Δ*pilB3-C3* after a 30-h incubation, suggesting that residual attachment of Δ*pilB3-C3* is not due to PilB1 and PilC1 driving assembly of PilA[1-6] pili (Figure 3A). Consistent with these results, as noted above, while *pilB1-C1his* expressed *in trans* can complement the adhesion defect of Δ*pilB1-C1*, *pilB1-C1his* expressed in a ∆*pilB3-C3* strain does not affect or recover the adhesion defect of this mutant strain. Therefore, taken together, these results suggest that PilB1-C1 cannot promote PilA[1–6] pilus biosynthesis and that the residual adhesion observed in Δ*pilB3-C3* depends upon another mechanism. It is possible that pilins within the membrane are sufficient to mediate the level of adhesion we observed in this strain, as previously suggested (Esquivel and Pohlschroder, 2014). Western blot analyses using anti-his antibodies of protein extracts from *H. volcanii* Δ*pilB3-C3* expressing *pilB1-C1his* in trans, unfortunately, did not reveal a PilChis band. Therefore, the possibility that it is not stably expressed cannot be ruled out. However, we also failed to detect a PilChis band in protein extracts from the Δ*pilB1-C1* background, where adhesion assays suggested that these proteins are expressed (data not shown).

### Analysis of PilB3-C3 Involvement in PilB1-C1-Dependent Adhesion

**Figure 5:**
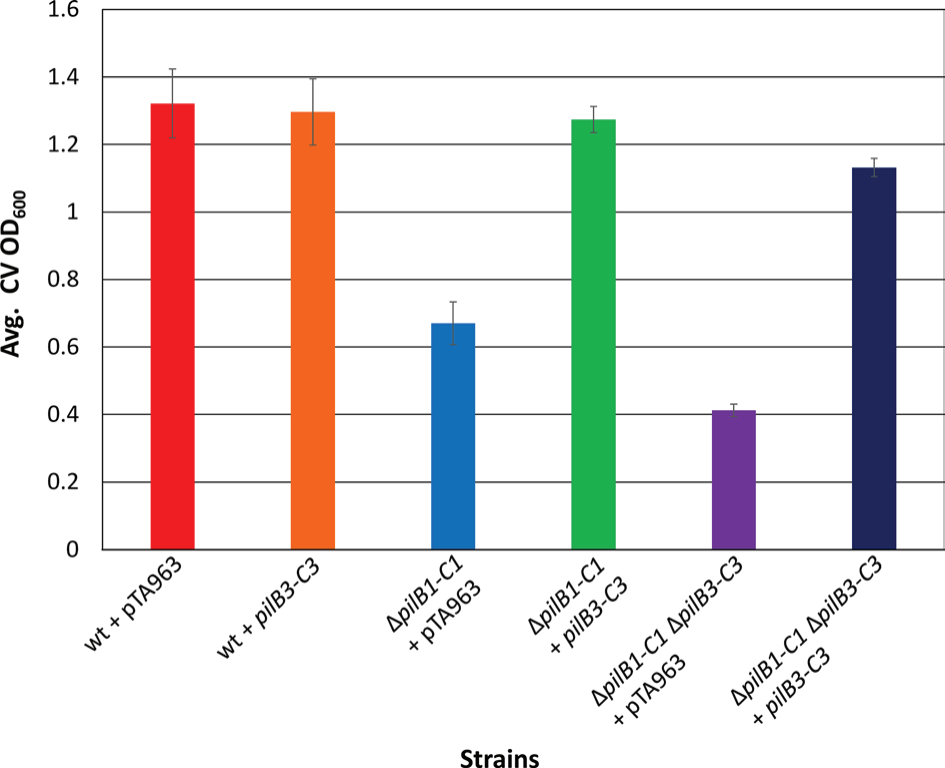
*pilB3-C3* expression *in trans* complements the Δ*pilB1-C1* and Δ*pilB1-C1* Δ*pilB3-C3* adhesion defects. Using the 96-well ALI assay, as described in Figure 3A, averages (± s.d.) of adhesion quantified through OD_600_ measurements of CV for three biological replicates with three technical replicates each, of wild-type, Δ*pilB1-C1*, and Δ*pilB1-C1* Δ*pilB3-C3* strains transformed with pTA963 and pTA963 encoding his-tagged *pilB3-C3* grown in CA media. It is shown that Δ*pilB1-C1* + *pilB3-C3* has similar adhesion levels to wild-type + pTA963 unlike Δ*pilB1-C1 +* pTA963.

To test the hypothesis put forth by Makarova et al. suggesting that the modular evolution of type IV pili, along with PilB and PilC, proteins required for their assembly, results in combinatorial variability of these protein complexes, we expressed *pilB3-C3his,* which was previously shown to complement the adhesion defect of a Δ*pilB3-C3* strain, in the Δ*pilB1-C1* background strains (Makarova et al., 2016). Consistent with this hypothesis, we determined that *pilB3-C3his* expression strongly complements the adhesion defects of these strains, increasing adhesion of the Δ*pilB1-C1* strain to a level similar to that of the pTA963-transformed wild-type strain and Δ*pilB1-C1*Δ*pilB3-C3* adhesion to nearly the same level (Figure 5). To exclude the possibility that PilB3-C3 might promote increased biosynthesis of PilA[1–6] pili, PilB3-C3his was expressed *in trans* in a wild-type strain, which might then be expected to attach to a surface more effectively. However, that is not the case, as the transformed wild-type strain adheres similarly to the strain transformed with pTA963 (Figure 5). Hence, these results suggest that PilB3-C3 can promote pilus formation of PilB1-C1-dependent pili.

## Concluding Remarks

The results of the deletion and complementation studies of *pilB* and *pilC* paralogs in the model archaeon, *H. volcanii,* presented in this study support the hypothesis posited by Makarova et al. that *pilB1-C1*, which phylogenetically clusters with type IV pilus biosynthesis genes of clade 4C, were acquired through lateral transfer from a crenarchaeal species, but are involved in assembling type IV pili composed, at least in part, of euryarchaeal type IV pilins belonging to clade 2. This was determined, at least in part, based on previous *in vivo* studies in *H. volcanii* that identified the major pilins of this clade demonstrating a complementary synergism of *in silico* and *in vivo* approaches for facilitating biological discoveries (Esquivel and Pohlschroder, 2014). Furthermore, these combined results might suggest future experiments for determining the mechanisms used by a distinct PilB-C paralog to recognize and assemble specific pilins and exclude others in the generation of a pilus perhaps allowing us to better understand the complexities involved in the biosynthesis of this universally conserved surface structure. While the adhesion defect of ∆*pilB1-C1* revealed by the transposon insertion mutant was invaluable for these studies, further testing may well reveal stronger, or weaker, phenotypes for this mutant under other conditions, underscoring the evolutionary advantage of the acquisition of an additional paralog by *H. volcanii* through lateral transfer. When expressed under certain conditions, these acquired paralogs might facilitate adhesion to additional abiotic surfaces, or to other cells. For example, in *Sulfolobus acidocaldarius,* UV-induced type IV pili promote cell aggregation leading to increased DNA exchange in this hyperthermophilic archaeon (Frols et al., 2008; Ajon et al., 2011). At least under the growth conditions used and with the abiotic surfaces tested, *pilB3-C3*, when constitutively expressed, can complement the phenotypic defects caused by a *pilB1-C1* deletion. Undoubtedly, the accumulation of additional transcriptomic and proteomic data for *H. volcanii*, as well as other euryarchaea containing these clades of type IV pili and pilins, will lead to a better understanding of the specific roles these PilB-C paralogs play in the cell surface biology of the euryarchaea.

## Author Contributions

GL and MP conceived and designed the experiments. GL performed the experiments involving screening, characterization of adhesion mutants, and adhesion assays. GL and MP analyzed the data. GL and MP wrote the manuscript.

### Conflict of Interest Statement

The authors declare that the research was conducted in the absence of any commercial or financial relationships that could be construed as a potential conflict of interest.
